# Old and new joint characterizations of leximin and variants of rank-weighted utilitarianism

**DOI:** 10.1371/journal.pone.0296351

**Published:** 2024-01-02

**Authors:** Norihito Sakamoto

**Affiliations:** Tokyo University of Science, Noda, Japan; Minnan Normal University, CHINA

## Abstract

This paper proposes a new class of efficient and equitable social welfare orderings, a *generalized leximin* rule that includes rank-weighted utilitarianism, leximin, and their lexicographic compositions. While the famous Deschamps and Gevers’ joint characterization theorem shows that a Paretian, anonymous, separable social welfare ordering must be either weak utilitarianism, leximin, or leximax under the assumption of cardinal full comparability, this study provides a new joint characterization theorem in which imposing rank-separability, instead of separability, enables acceptable social welfare ordering to be the generalized leximin rule. This result is proven by an intuitive and easy-to-understand method, which also helps show the mechanism by which a class of Paretian, anonymous, and separable social welfare orderings is limited to weak utilitarianism and leximin.

## Introduction

In all public value judgments such as economic evaluations of public health, infrastructure planning, redistributive taxation, and welfare policies to reduce poverty, there is a need to aggregate individual preferences and well-being to assess social welfare. However, given a specific measure of individual well-being, how should well-being be aggregated to obtain consistent social value judgments? What aggregation method is preferable, according to the degree of intrapersonal or interpersonal comparability of individual well-being?

While Arrow’s impossibility theorem [1] shows that no aggregation rule satisfies reasonable properties under the assumption of ordinal non-comparability of utilities, Sen [2, 3] shows that admitting interpersonal comparisons of utility changes this theorem to achieve some possibility results. Many possibility theorems have been successfully achieved by several studies [4–17]. They show the following: (i) the leximin rule is the only social welfare ordering that satisfies plausible axioms when ordinal-level comparability is admissible [2, 4]; (ii) several utilitarian-type social welfare orderings are acceptable candidates when cardinal partial comparability is admissible [6, 10, 13]; and (iii) either the weak utilitarian or leximin rules can survive when cardinal full comparability is admissible [8].

Do the results discussed in the literature indicate that a class of acceptable social welfare orderings under the assumption of cardinal full comparability is limited to only two rules–utilitarianism or leximin? A well-known flaw in utilitarianism is that there is no consideration for distribution when comparing social welfare. The rule considers only the sum of the individual utilities. However, the major drawback of leximin is that it ignores overall welfare loss and cannot satisfy continuity because of excessive consideration for distribution. An aggregation method that lies between these extreme rules is the so-called *generalized Gini inequality index* (rank-weighted utilitarianism) proposed by Weymark [18]. According to this aggregation method, social welfare is defined as a rank-dependent weighted sum of well-being in which higher weights are assigned to lower well-being. Unlike a simple utilitarian rule, it can satisfy not only equity axioms in distributive justice but also continuity. Hence, abandoning separability allows us to find possibilities for distribution-sensitive social welfare orderings under the assumption of cardinal full comparability. Indeed, this study shows the whole picture of a broad class of distribution-sensitive social welfare orderings: the *generalized leximin* rule, which includes leximin, the above Gini rules, and their lexicographic compositions (e.g. the utilitarian-first and leximin-second rule, the leximin-first and utilitarian-second rule, and the quantile mean comparison method). Moreover, by requiring *rank separability* instead of separability, this study succeeds in providing a new and generalized version of Deschamps and Gevers’ joint characterization theorem, in which a class of acceptable rules must be generalized leximin.

The generalized leximin rule is defined as follows. First, the set of individuals is divided into subgroups according to their well-being ranks from bottom to top. Next, each weighted sum of well-being for each subgroup is calculated. Then, the rule lexicographically judges well-being profiles following the sequences of the weighted sum of well-being, as defined above. If all the subgroups are singletons, it is equivalent to leximin. If the subgroup is an entire set of individuals, it is equivalent to a refinement of the generalized Gini inequality index. Thus, the generalized leximin rule is a broad class of distribution-sensitive social welfare orderings that can include the leximin rule, the generalized Gini inequality index, and various orderings as their lexicographic compositions. The proof of this theorem is elementary and intuitive, and is also useful for proving the old joint characterization theorem.

The study makes two main contributions to the literature. First, it discovers and characterizes a new class of distribution-sensitive social welfare orderings, the generalized leximin rule. Second, it provides an elementary proof that makes it easier to understand how each axiom works in related axiomatic characterizations.

The remainder of this paper is organized as follows. The next two sections describe the basic notations and definitions, and then define the various social welfare orderings that are axiomatically characterized in this study. The old and new characterization results are then presented, and the final section discusses future research issues before concluding the paper.

## Basic definitions

This section explains the basic notation, definitions, and axioms used in this study. Let *N* = {1, …, *n*} be a set of individuals with *n*≥3. The set of all possible well-being profiles is denoted by *U* ^*N*^. Suppose *U* ^*N*^ = ℝ^*N*^. I use the following notation ≧ and > for vector inequalities. For all *u*_*N*_, *v*_*N*_ ∈*U* ^*N*^, *u*_*N*_≧*v*_*N*_ iff *u*_*i*_≥*v*_*i*_ for all *i*, and *u*_*N*_>*v*_*N*_ iff *u*_*i*_≥*v*_*i*_ for all *i* and *u*_*j*_>*v*_*j*_ for some *j*. For all *u*_*N*_ ∈*U* ^*N*^, let *u*_[*N*]_ = (*u*_[*1*]_, *u*_[*2*]_, …, *u*_[*n*]_) be a non-decreasing rearrangement of *u*_*N*_; that is, *u*_[1]_≤*u*_[2]_≤⋯≤*u*_[*n*]_. The set of ranks is denoted as [*N*] = {[1], [2], …, [*n*]}. Given *u*_*N*_ ∈*U* ^*N*^, for any subset *M* of *N*, let *u*_*M*_ and *u*_−*M*_ be (*u*_*i*_)_*i*∈*M*_ and (*u*_*i*_)_*i*∈*N*\*M*_, respectively. In the similar way, for any subset [*M*] of [*N*], let *u*_[*M*]_ and *u*_−[*M*]_ be (*u*_[*i*]_)_[*i*]∈[*M*]_ and (*u*_[*i*]_)_[*i*]∈[*N*]\[*M*]_, respectively. For an arbitrary non-empty set *X*, a binary relation defined on *X* is an *ordering* if and only if it satisfies completeness and transitivity. Completeness requires that, for all *x*, *y* in *X*, *x*≽*y* or *y*≽*x*. Transitivity requires that, for all *x*, *y*, *z* in *X*, *x*≽*y* & *y*≽*z* implies *x*≽*z*. Let a *social welfare ordering* ≽ be defined on *U* ^*N*^. For all *u*_*N*_, *v*_*N*_ ∈*U* ^*N*^, *u*_*N*_≽*v*_*N*_ means that *u*_*N*_ is at least as socially good as *v*_*N*_. The asymmetric and symmetric parts of the social welfare ordering ≽ are given by ≻ and ~, respectively. That is, for all ∀*u*_*N*_, *v*_*N*_∈*U*^*N*^, *u*_*N*_≻*v*_*N*_↔*u*_*N*_≽*v*_*N*_ and *not v*_*N*_≽*u*_*N*_, and *u*_*N*_~*v*_*N*_↔*u*_*N*_≽*v*_*N*_ and *v*_*N*_≽*u*_*N*_.

To define distributive equity axioms that require certain types of interpersonal comparisons of well-being, each individual’s well-being is assumed to be cardinal and fully comparable. Following the tradition of social choice theory, I require the following invariance condition. This invariance axiom requires that social ranking should be the same with respect to any positive affine transformation of well-being.

### Cardinal full comparability

$\forall u_{N},v_{N}\in U^{N},\forall a\in R,\forall b\in R_{++},u_{N}≽v_{N}↔(a+bu_{i}{)}_{i\in N}≽(a+bv_{i}{)}_{i\in N}$.

However, as Morreau and Weymark [19] correctly point out, such a kind of invariance axioms imply not interpersonal comparability of utilities but just scale invariance, which drastically restricts functional forms of social welfare orderings.

Next, let us define a series of axioms that should be satisfied by acceptable social welfare orderings. First, as an axiom of efficiency, the strong Pareto principle is required. This axiom demands that social welfare should increase whenever no one’s well-being level falls and at least one increases.

### Strong Pareto

∀*u*_*N*_, *v*_*N*_∈*U*^*N*^, if *u*_*N*_≧*v*_*N*_, then *u*_*N*_≽*v*_*N*_. Moreover, if *u*_*N*_>*v*_*N*_, then *u*_*N*_≻*v*_*N*_.

Throughout the paper, I assume that all social welfare orderings must treat each individual’s well-being impartially, and this requirement is the following *anonymity* axiom.

### Anonymity

∀bijections *π* on *N*, ∀*u*_*N*_∈*U*^*N*^, *u*_*N*_~*u*_*π*(*N*)_.

Some results may require *continuity* while the main result does not need this property. This axiom says that any small change in a profile never yields a big change in its social ranking. Formally, this demands that both the upper and lower contour sets of the social welfare ordering be closed.

### Continuity

∀*u*_*N*_∈*U*^*N*^, the sets {*v*_*N*_∈*U*^*N*^|*u*_*N*_≽*v*_*N*_} and {*v*_*N*_∈*U*^*N*^|*v*_*N*_≽*u*_*N*_} are closed in ℝ^*N*^.

*Separability* requires social welfare orderings to ignore the information of well-being about indifferent (i.e., unaffected) individuals between the two profiles. As shown in the proof of Theorem 3, this axiom plays a central role in the famous joint characterization theorem, in which Paretian and anonymous social welfare orderings must be weak utilitarian, leximin, or leximax.

### Separability

$\forall u_{N},v_{N},u_{N}^{\prime },v_{N}^{\prime }\in U^{N}$, if $\exists M\subseteq N,(u_{M}=u_{M}^{\prime }\&v_{M}=v_{M}^{\prime })\&(u_{-M}=v_{-M}\&u_{-M}^{\prime }=v_{-M}^{\prime })$, then $u_{N}≽v_{N}↔u_{N}^{\prime }≽v_{N}^{\prime }$.

Despite the advantage of simple additivity, separability may have some problems in the sense that it cannot consider relative inequality in distributive justice. For example, consider the following well-being profiles: (10, 20, 90, 100), and (8, 30, 90, 100). In this case, the third and fourth individuals are indifferent among the two profiles. Then, separability requires that the ranking of the two profiles (10, 20, 90, 100) and (8, 30, 90, 100) be the same on (10, 20, 1, 0) and (8, 30, 1, 0), which may not seem plausible for those who care about relative inequality. To see this, suppose that the society give some priority for the worst individual’s well-being (say, the weight of the worst is 0.7, and the other’s weight is 0.1). Then, the generalized Gini index prefers (10, 20, 90, 100) to (8, 30, 90, 100) while it prefers (8, 30, 1, 0) to (10, 20, 1, 0). Therefore, let us weaken separability to the following *rank separability*.

### Rank separability

$\forall u_{N},v_{N},u_{N}^{\prime },v_{N}^{\prime }\in U^{N}$, if $\exists [M]\subseteq [N],(u_{\left[M\right]}=u_{\left[M\right]}^{\prime }\&v_{\left[M\right]}=v_{\left[M\right]}^{\prime })\&(u_{-[M]}=v_{-[M]}\&u_{-[M]}^{\prime }=v_{-[M]}^{\prime })$, then $u_{N}≽v_{N}↔u_{N}^{\prime }≽v_{N}^{\prime }$.

This axiom requires social welfare orderings to ignore information about the same value of well-being in the same ranks between the two profiles. Separability implies rank separability whenever anonymity is required. Note that rank separability implies anonymity [20]. The study demonstrates later that simply imposing rank separability instead of separability yields a broad class of distribution-sensitive social welfare orderings.

Finally, I introduce the well-known equity axiom often used in income inequality measurements. *Pigou-Dalton transfer* requires that for any profile, a transfer that reduces the well-being gap with keeping the total sum of the profile does not decrease social welfare. This requirement seems plausible if each well-being is fully interpersonal comparable and there is no legitimate ethical reason for the gap to remain.

### Pigou-Dalton transfer

$\forall u_{N},v_{N}\in U^{N},\forall \varepsilon \in R_{++}$, if $\exists i,j\in N,v_{i}-\varepsilon =u_{i}\geq u_{j}=v_{j}+\varepsilon$ and $\forall k\in N\setminus \{i,j\},u_{k}=v_{k}$, then *u*_*N*_≽*v*_*N*_.

## Social welfare orderings

This section introduces various social welfare orderings, which are characterized in the next section. The following rules satisfy standard axioms: cardinal full comparability, strong Pareto, anonymity, and separability (or rank separability). First, I introduce three social welfare orderings: the weak utilitarian, leximin, and leximax rules, jointly characterized by the axioms of anonymity, strong Pareto, separability, and cardinal full comparability in Theorem 3.

The weak utilitarian rule judges social welfare by summing individual well-being. If a sum of well-being in a profile *u*_*N*_ is higher than a sum in *v*_*N*_, then the weak utilitarian rule strictly prefers *u*_*N*_ to *v*_*N*_. Note that this rule says nothing about a situation in which total sums are the same between two profiles.

### Weak utilitarianism

A social welfare ordering ≽^*WU*^ is *weak utilitarian* if and only if $\forall u_{N},v_{N}\in U^{N},\sum_{i\in N}u_{i}>\sum_{i\in N}v_{i}{\to u}_{N}{≻}^{WU}v_{N}$

As d’Aspremont and Gevers [5] and Sen [2] show, the leximin and leximax rules can be jointly characterized in the setting of ordinal-level comparability (co-ordinality).

### Leximin

A social welfare ordering ≽^*LM*^ is *leximin* if and only if $\forall u_{N},v_{N}\in U^{N},u_{N}{≽}^{LM}v_{N}↔[\forall [i],u_{\left[i\right]}=v_{\left[i\right]}]$ or $\left[\exists [j],{\forall [i]<[j],u}_{\left[i\right]}=v_{\left[i\right]}\&u_{\left[j\right]}>v_{\left[j\right]}\right]$.

The leximin rule judges social welfare by following a lexicographic ordering that sequentially evaluates a hierarchy of well-being from the bottom to the top. The leximin rule has various axiomatic characterizations [2]. The reversed version of leximin is the *leximax* rule, which is usually interpreted as unacceptable from the viewpoint of distributive justice.

### Leximax

A social welfare ordering ≽^*LX*^ is *leximax* if and only if $\forall u_{N},v_{N}\in U^{N},u_{N}{≽}^{LX}v_{N}↔[\forall [i],u_{\left[i\right]}=v_{\left[i\right]}]$ or $\left[\exists [j],{\forall [i]>[j],u}_{\left[i\right]}=v_{\left[i\right]}\&u_{\left[j\right]}>v_{\left[j\right]}\right]$.

As Dechamps and Gevers [8] show, these three rules are jointly characterized by the standard axioms under the assumption of cardinal full comparability. On the other hand, as Ebert [20] shows, the following rank-weighted utilitarian rule is characterized by the axioms of strong Pareto, rank separability, continuity, and cardinal full comparability (See Theorem 2).

### Rank-weighted utilitarianism

A social welfare ordering ≽^*RU*^ is *rank-weighted utilitarian* if and only if ∃*w*_[*N*]_∈(0, 1)^*N*^ with $w_{\left[1\right]}\geq \cdots \geq w_{\left[n\right]}\&\sum_{\left[i]\in [N\right]}w_{\left[i\right]}=1,\forall u_{N},v_{N}\in U^{N},u_{N}{≽}^{RU}v_{N}↔\sum_{\left[i]\in [N\right]}w_{\left[i\right]}u_{\left[i\right]}\geq \sum_{\left[i]\in [N\right]}w_{\left[i\right]}v_{\left[i\right]}$.

Rank-weighted utilitarianism was proposed by Weymark [18] and is a generalization of social welfare orderings based on the Gini coefficient. This rule measures social welfare using a rank-dependent weighted sum of well-being, in which each weight is assigned to each rank. Also, the rank-weighted utilitarian rule can be easily defined on a set of profiles focusing on a subset of specific ranks. For example, for a set of ranks [*M*] = {[4], [5], [6]}, a value function of a profile *u*_[*M*]_ can be defined by $\sum_{\left[i]\in [M\right]}w_{\left[i\right]}u_{\left[i\right]}$. To define the generalized leximin rule, I will use this extended version of the rank-weighted utilitatian rule defined on *U*^[*M*]^.

Finally, let’s introduce the generalized leximin rule which will be characterized in Theorem 1. Intuitively, the generalized leximin rule compares profiles by following a lexicographic ordering defined on a sequence of weighted sums of subgroups’ well-being. Each subgroup is composed of sequential numbers of ranks, and no subgroup exists in which the rank number is not adjacent to others.

As a preliminary step of the definition, a partition ([*M*_*1*_], …, [*M*_*k*_]) of [*N*] is called a *rank-hierarchical partition* if and only if for all natural numbers *i*, *j* with *i* < *j*, for all ranks [*k*_*i*_]∈[*M*_*i*_], and for all ranks [*k*_*j*_]∈[*M*_*j*_], [*k*_*i*_] < [*k*_*j*_] holds. That is, a rank-hierarchical partition is a sequence of divided subsets of adjacent ranks from bottom to top. Also, for all binary relations ≽, ≽* defined on X, ≽* is a refinement of ≽ if and only if for all *x*, *y* in *X*, *x*≻*y* implies *x*≻*y. By using these definitions, the *generalized leximin* rule is defined as follows.

### Generalized leximin

A social welfare ordering ≽^*GL*^ is *generalized leximin* if and only if ∃a rank-hierarchical partition ([*M*_*1*_], …, [*M*_*k*_]) of [*N*], ∃ a sequence of orderings ${(≽}_{\left[M1\right]}^{RU*},\ldots ,{≽}_{\left[M_{k}\right]}^{RU*})$, $\forall u_{N},v_{N}\in U^{N},u_{N}{≽}^{GL}v_{N}↔[\forall i\in \{1,\ldots ,k\},\;u_{\left[M_{i}\right]}{∼}_{\left[M_{i}\right]}^{RU*}v_{\left[M_{i}\right]}]\;or\;[\exists j\in \{1,\ldots ,k\},\forall i<j,\;u_{\left[M_{i}\right]}{∼}_{\left[M_{i}\right]}^{RU*}v_{\left[M_{i}\right]}\;and\;u_{\left[M_{j}\right]}{≻}_{\left[M_{j}\right]}^{RU*}v_{\left[M_{j}\right]}]$, where $\forall i\in \{1,\ldots ,k\},{≽}_{\left[M_{i}\right]}^{RU*}$ is a refinement of the rank weighted utilitarian rule defined on the domain *U*^[*Mi*]^.

As the definition clearly states, if the subgroups are all singletons, then the generalized leximin rule must be the leximin rule. If the subgroups to the specific group are all singletons and the remaining subgroup is the whole complement of them, then it must be the leximin-first and rank-weighted utilitarian-second rule which is a lexicographic composition of the leximin and the rank-weighted utilitarian rules. This rule, which is unknown in the literature, always follows the leximin rule up to a fixed rank. Whenever two profiles have the same well-being levels up to this rank, it simply follows the rank-weighted utilitarian rule. If the subgroups are simply some quantiles, it must be a refinement of the quantile mean comparison method which is proposed by Sakamoto and Mori [21]. Furthermore, if the subgroup is equal to the entire set of individuals, then it must be a refinement of the rank-weighted utilitarian rule. That is, this refinement includes rank-weighted utilitarianism, utilitarianism, the rank-weighted utilitarian-first and leximin-second rule, and the utilitarian-first and leximin-second rule. Hence, this rule can be interpreted as a generalization of the entire class of distribution-sensitive rules that satisfy rank separability.

## Old and new joint characterizations

This section shows that the generalized leximin rule is implied by strong Pareto, Pigou-Dalton transfer, rank-separability (implying anonymity), and cardinal full comparability. By dropping separability and requiring rank-separability, policy makers obtain a broad class of Paretian, anonymous, and distribution-sensitive rules, including leximin, rank-weighted utilitarianism, and their lexicographic compositions. The proof of this result can be completed by several elementary claims, which can facilitate our understanding of the reasons why Deschamps and Gevers’ joint characterization theorem holds. First, let us show a new joint characterization theorem for the generalized leximin rule in a reduced form.

### Theorem 1

If a social welfare ordering satisfies strong Pareto, rank separability, Pigou-Dalton transfer, and cardinal full comparability, then it is generalized leximin.

As Tungodden [22] and Tungodden and Vallentyne [23] show, combining strong Pareto and perfect equity (see S2 Appendix on its formal definition) implies that a social welfare ordering must be a refinement of the maximin rule. If perfect equity is added to the axioms of Theorem 1, then [*M*_1_] must be [1] in a partition of the generalized leximin rule; that is, the rule must be a refinement of the maximin rule.

Gevers [9] showed that combining strong Pareto, anonymity, and *almost co-cardinality* (see S2 Appendix on its formal definition) implies that a social welfare ordering must be a refinement of rank-weighted utilitarianism with zero rank-dependent weights of some combination of ranks. Almost co-cardinality is a mixed axiom of *level- and gain-*comparability in well-being, which is consistent with a very limited version of separability. If this rank-weighted utilitarianism gives zero weight to all ranks except for a single rank, then this ordering is a refinement of the well-known rank dictatorship [11, 12]. Note that the generalized leximin rule can be interpreted as a subclass of Gevers’ refinement.

The generalized leximin rule does not always prioritize individuals with lower well-being despite the definition of a descending weight vector assigned to ranks. For example, consider the following rule. In a three-individual economy, the rule lexicographically judges the following sequence of weighted sums in the two subgroups: (∑_*i* = 1, 2_ *w*_[*i*]_*u*_[*i*]_, ∑_*i* = 3_ *u*_[*i*]_). Furthermore, this rule prefers a profile with a lager rank 2’s well-being to that with a smaller rank 2’s well-being whenever the sum of ranks 1and 2’s well-being is the same. To avoid this problem, a stronger equity axiom is required.

Next, if I add continuity to the system of axioms stated above, the generalized leximin rule must be rank-weighted utilitarian. This result was first shown by Ebert [20], who used functional analysis techniques. However, because the only continuous generalized leximin rule is obviously rank-weighted utilitarian, it is easy to understand and prove Ebert’s result.

### Theorem 2

A social welfare ordering satisfies strong Pareto, rank-separability, Pigou-Dalton transfer, continuity, and cardinal full comparability, if and only if it is rank-weighted utilitarian.

Let us consider a class of Paretian and anonymous social welfare orderings that satisfy separability instead of rank separability. From Claim 1 in the proof of Theorem 1, it can be easily understood that each rank’s threshold of well-being is constant regardless of rank under the separability requirement. Indeed, when separability is required, possible transfer thresholds defined for any rank’s well-being are limited to 1, 0.5, or 0 to ensure a constant threshold for all ranks. If the threshold equals 1, then it is leximax. If the threshold is 0.5, then it is weakly utilitarian. If the threshold equals 0, then it is leximin. Hence, I easily prove Deschamps and Gevers’ joint characterization theorem using the above facts and the proof of Theorem 1.

### Theorem 3

If *a social welfare ordering satisfies strong Pareto*, *anonymity*, *separability*, *and cardinal full comparability*, *then it is weak utilitarian*, *leximin*, *or leximax*.

Weak utilitarianism may ignore distributive justice because it could be the *utilitarian-first and leximax-second* rule. Of course, both the utilitarian and the utilitarian-first and leximin-second rules belong to a class of the weak utilitarian rules. Hence, weak utilitarianism could be slightly distribution-sensitive in the form of a subsidiary criterion of the leximin rule. Kamaga [24] shows that the leximin and the utilitarian-first and leximin-second rules are jointly characterized by two additional equity axioms: strict Pigou-Dalton transfer and strict composite transfer (see S2 Appendix on these formal definitions).

In Theorem 3, separability rules out the leximin-first and utilitarian-second rule because it ignores rank information, and it cannot allow the well-being transfer of different ranks to have different impacts on social welfare. As a result, whenever separability is required, a class of distribution-sensitive rules is either the leximin or the utilitarian-first and leximin-second rules (that is, the very weak distribution-sensitive rule). Hence, if a strict version of minimal equity is imposed on a social welfare ordering, leximin is the only option for acceptable rules. For example, consider the following minimal equity requirement.

### Minimal transfer equity

$\exists u_{N},v_{N}\in U^{N},\exists \varepsilon \in R_{++},\exists i,j\in N,v_{i}-\varepsilon =u_{i}\geq u_{j}=v_{j}+\varepsilon ,\;\forall k\in N\setminus \{i,j\},\;u_{k}=v_{k},$ and *u*_*N*_≻*v*_*N*_.

Combining this minimal equity condition and Theorem 3 leads to the following result immediately.

### Theorem 4

A social welfare ordering satisfies strong Pareto, anonymity, separability, minimal transfer equity, and cardinal full comparability, if and only if it is leximin.

Finally, adding continuity to the axioms required in Deschamps and Gevers [8] eliminates the possibility of leximin and leximax, leaving only the utilitarian rule. Old and new joint characterization theorems indicate that there is no binary choice between utilitarianism (no consideration for well-being distribution) and leximin (excess consideration for distribution and few interests in total welfare) in seeking desirable aggregation methods that satisfy cardinal full comparability. Furthermore, showing that a class of acceptable social welfare orderings can be characterized by the generalized leximin rule, which is a broad class of distribution-sensitive rules, the central doctrine of social welfare can be simply interpreted as determining *tolerable inequality*.

## Conclusion

This study shows that a class of acceptable social welfare orderings with rank separability is the generalized leximin rule. By providing an elementary proof method, this study also succeeds in proving the old joint characterization theorem, in which a class of Paretian, anonymous, and separable social welfare orderings must be either weak utilitarianism, leximin, or leximax.

Several issues remain that further research can address. First, this study finds a variety of distribution-sensitive social welfare orderings using rank separability. However, a question remains: what rules would be acceptable if there were no requirements for rank separability? Based on the proof of this study, if rank separability is not required, each rank’s weight differs in each profile of individual well-being. In other words, given a profile of individual well-being, a class of Paretian, anonymous, and *continuous* social welfare orderings must belong to the class of rank-weighted utilitarianism with variable rank-dependent weights. However, what types of rank-dependent weight vectors have legitimacy and consistency? A new persuasive axiom is required to ensure acceptable social welfare ordering.

Second, by dropping the cardinal full comparability, I may obtain a new version of the joint characterization theorem. As is well known, generalized utilitarianism is characterized by the following standard axioms: strong Pareto, anonymity, Pigou-Dalton transfer, continuity, and separability. Thus, it is expected that a class of acceptable social welfare orderings must be either *a lexicographic combination of weak generalized utilitarianism with disjoint intervals* or leximin. If I require some scale invariance conditions such as the so-called cardinal unit comparability and cardinal ratio comparability, then its functional form can be specified by Kolm-Pollack or Atkinson type just like the very famous characterization results in Blackorby and Donaldson [13] and Ebert [25].

Finally, in using the generalized leximin rule to measure social welfare, a social planner must solve a practical problem regarding how to determine an acceptable set of rank-dependent weight vectors, that is, the level of *tolerable inequality* in a society. This problem cannot be determined by any axiomatic analysis, but by a political process of a compromised consensus across a society, which is sometimes unfair for social minorities and vulnerable groups. This may be solved by analyzing normative behaviors and reactions of human beings in well-designed empirical or experimental situations, which seem to be determined by emotional and negative reactions to inequality, oppression, and injustice in fair and informative circumstances.

## Supporting information

S1 Appendix The proof of Theorem 1: The proof can be completed by using the following five claims. (DOCX)

S2 Appendix This appendix provides the definitions of the axioms that are not formally explained in the paper [22, 26]. (DOCX)
