# Supplementary material for: Old and new joint characterizations of leximin and variants of rank-weighted utilitarianism
Source: PLoS One. 2024 Jan 2;19(1):e0296351. doi: 10.1371/journal.pone.0296351 (PMC10760868; doi:10.1371/journal.pone.0296351)
Supplement: S1 Appendix — (DOCX) [file pone.0296351.s001.docx]

**Appendix 1.**

The Proof of Theorem 1: The proof can be completed by using the following five claims.

(Claim 1)∀[*i*] ≠ [1], ∃! *α*_[_*_i_*_]_∈[0, 1/2], ∀*u_N_* ∈*U ^N^* with *u*_[_*_i_*_]_ > *u*_[_*_i-1_*_]_,∀*α' >α*_[_*_i_*_]_, (*α' u*_[_*_i_*_]_ + (1 - *α'*)*u*_[_*_i-1_*_]_ , *α' u*_[_*_i_*_]_ + (1 - *α'*)*u*_[_*_i-1_*_]_ , *u*_-[_*_i-1_*_,_ *_i_*_]_) ≻ *u_N_* and ∀*α' <α*_[_*_i_*_]_, *u_N_* ≻ (*α' u*_[_*_i_*_]_ + (1 - *α'*)*u*_[_*_i-1_*_]_ , *α' u*_[_*_i_*_]_ + (1 - *α'*)*u*_[_*_i-1_*_]_, *u*_-[_*_i-1_*_,_ *_i_*_]_), where the notation *u*_-[_*_i-1_*_,_ *_i_*_]_ is *u*_-{[_*_i-1_*_], [_*_i_*_]}_.

For all *α*∈[0, 1], let *u*_[_*_N_*_]_(*α*) be denoted by the profile (*α u*_[_*_i_*_]_ + (1 - *α*)*u*_[_*_i-1_*_]_ , *α u*_[_*_i_*_]_ + (1 - *α*)*u*_[_*_i-1_*_]_ , *u*_-[_*_i-1_*_,_ *_i_*_]_). By strong Pareto and rank separability (which implies anonymity), *u*_[_*_N_*_]_(1) ≻ *u_N_* ≻ *u*_[_*_N_*_]_(0). Completeness implies that *u*_[_*_N_*_]_(*α*) ≻ *u_N_* or *u_N_* ≽ *u*_[_*_N_*_]_(*α*) for all *α*∈[0, 1]. Also, strong Pareto implies that *u*_[_*_N_*_]_(*α*) is strictly increasing w. r. t. *α*, that is, ∀*α', α''* with *α' >α''*, *u*_[_*_N_*_]_(*α'*) ≻ *u*_[_*_N_*_]_(*α''*). Thus, one can determine a threshold *α*_[_*_i_*_]_ in [0, 1] by decreasing *α* from 1 to 0 in *u*_[_*_N_*_]_(*α*). Moreover, Pigou-Dalton transfer guarantees that a threshold *α*_[_*_i_*_]_ must be in [0, 1/2]. This fact is easily shown as follows. Suppose a threshold *α*_[_*_i_*_]_ >1/2. By the definition of threshold and strong Pareto, for all *α'* in (1/2, *α*_[_*_i_*_]_), *u_N_* ≻ *u*_[_*_N_*_]_(*α'*) ≻ *u*_[_*_N_*_]_(1/2). However, Pigou-Dalton transfer implies *u*_[_*_N_*_]_(1/2) ≽ *u_N_*. A contradiction.

Next, I demonstrate the uniqueness of threshold *α*_[_*_i_*_]_. For all *u_N_*, *v_N_* ∈*U ^N^* with *u*_[_*_i_*_]_ > *u*_[_*_i-1_*_]_, let *a* = (*v*_[_*_i_*_]_ *- v*_[_*_i-1_*_]_)(*u*_[_*_i_*_]_ *- u*_[_*_i-1_*_]_)^-1^ and *b* = [*v*_[_*_i-1_*_]_(*u*_[_*_i_*_]_ *- u*_[_*_i-1_*_]_) - *u*_[_*_i-1_*_]_(*v*_[_*_i_*_]_ *- v*_[_*_i-1_*_]_)](*u*_[_*_i_*_]_ *- u*_[_*_i-1_*_]_)^-1^. Then, *au*_[_*_i-1_*_]_ + *b* = *v*_[_*_i-1_*_]_ and *au*_[_*_i_*_]_ + *b* = *v*_[_*_i_*_]_. Note that both *u*_-[_*_i-1_*_,_ *_i_*_]_ and *v*_-[_*_i-1_*_,_ *_i_*_]_ can be any well-being vectors. This means that for all *v*_-[_*_i-1_*_,_ *_i_*_]_, there exists *u*_-[_*_i-1_*_,_ *_i_*_]_ such that for all ranks [*j*]≠[*i*] and [*i*-1], *au*_[_*_j_*_]_ + *b* = *v*_[_*_j_*_]_. Also, rank separability guarantees the threshold *α*_[_*_i_*_]_ in *u_N_* with *u*_[_*_i_*_]_ > *u*_[_*_i-1_*_]_ must be the same one in *u'_N_* with *u*_[_*_i_*_]_ = *u'*_[_*_i_*_]_ and *u*_[_*_i-1_*_]_ = *u'*_[_*_i-1_*_]._ Hence, these facts and cardinal full comparability guarantee the uniqueness of the threshold *α*_[_*_i_*_]_. ■

(Claim 2) ∀[*i*] ≠ 1, ∀*u_N_*, *v_N_* ∈*U ^N^* with *u*_-[_*_i-1_*_,_ *_i_*_]_ = *v*_-[_*_i-1_*_,_ *_i_*_]_, if *α*_[_*_i_*_]_ *u*_[_*_i_*_]_ + (1 -*α*_[_*_i_*_]_) *u*_[_*_i-1_*_]_ > *α*_[_*_i_*_]_ *v*_[_*_i_*_]_ + (1 - *α*_[_*_i_*_]_)*v*_[_*_i-1_*_]_ , then *u_N_* ≻ *v_N_*.

By the definition of threshold *α*_[_*_i_*_]_ in Claim 1 and strong Pareto, [∀*u'* with *u' > α*_[_*_i_*_]_*u*_[_*_i_*_]_ + (1 - *α*_[_*_i_*_]_)*u*_[_*_i-1_*_]_, (*u'*, *u'*, *u*_-[_*_i-1_*_,_ *_i_*_]_) ≻ *u_N_*] and [∀*u'* with *u' < α*_[_*_i_*_]_*u*_[_*_i_*_]_ + (1 - *α*_[_*_i_*_]_)*u*_[_*_i-1_*_]_, *u_N_* ≻ (*u'*, *u'*, *u*_-[_*_i-1_*_,_ *_i_*_]_)]. Since *α*_[_*_i_*_]_ *u*_[_*_i_*_]_ + (1 -*α*_[_*_i_*_]_) *u*_[_*_i-1_*_]_ > 0.5*α*_[_*_i_*_]_ (*u*_[_*_i_*_]_ + *v*_[_*_i_*_]_ ) + 0.5(1 -*α*_[_*_i_*_]_) (*u*_[_*_i-1_*_]_ + *v*_[_*_i-1_*_]_ ) > *α*_[_*_i_*_]_ *v*_[_*_i_*_]_ + (1 - *α*_[_*_i_*_]_)*v*_[_*_i-1_*_]_, I have *u_N_* ≻ (0.5*α*_[_*_i_*_]_ (*u*_[_*_i_*_]_ + *v*_[_*_i_*_]_ ) + 0.5(1 -*α*_[_*_i_*_]_) (*u*_[_*_i-1_*_]_ + *v*_[_*_i-1_*_]_ ), 0.5*α*_[_*_i_*_]_ (*u*_[_*_i_*_]_ + *v*_[_*_i_*_]_ ) + 0.5(1 -*α*_[_*_i_*_]_) (*u*_[_*_i-1_*_]_ + *v*_[_*_i-1_*_]_ ), *u*_-[_*_i-1_*_,_ *_i_*_]_) ≻ *v_N_*. Transitivity implies that *u_N_* ≻ *v_N_*. ■

From Claims 1 and 2, it turns out that the value *α*_[_*_i_*_]_ *u*_[_*_i_*_]_ + (1 -*α*_[_*_i_*_]_) *u*_[_*_i-1_*_]_ indicates a representative welfare level of two people’s well-being (*u*_[_*_i-1_*_]_, *u*_[_*_i_*_]_). Let us call a unique number *α*_[_*_i_*_]_ a *threshold* of [*i*], and a representative welfare level *α*_[_*_i_*_]_ *u*_[_*_i_*_]_ + (1 -*α*_[_*_i_*_]_) *u*_[_*_i-1_*_]_ *a critical value* of [*i-1*, *i*] in *u_N_*. For all profiles *u_N_*, *v_N_* ∈*U ^N^* with *u*_-[_*_i-1_*_,_ *_i_*_]_ = *v*_-[_*_i-1_*_,_ *_i_*_]_, the notation *u_N_* ≈_[_*_i_*_-1,_ *_i_*_]_ *v_N_* denotes that both *u_N_* and *v_N_* have the same critical value of [*i-1*, *i*]. Using the above notation, Claim 3 is as follows.

(Claim 3) ∀*u_N_* ∈*U ^N^*, ∀[*i*] ≠ [1] with its threshold *α*_[_*_i_*_]_ ≠ 0, ∀sufficiently small numbers *ε* > 0, *u_N_* ≈_[_*_i_*_-1,_ *_i_*_]_ (*u*_[_*_i-1_*_]_ + $\frac{\alpha_{[i]}}{1 - \alpha_{[i]}}$*ε*, *u*_[_*_i_*_]_ - *ε*, *u*_-[_*_i-1_*_,_ *_i_*_]_) ≈_[_*_i_*_-1,_ *_i_*_]_ (*u*_[_*_i-1_*_]_ - $\frac{\alpha_{[i]}}{1 - \alpha_{[i]}}$*ε*, *u*_[_*_i_*_]_ + *ε*, *u*_-[_*_i-1_*_,_ *_i_*_]_).

Claim 2 guarantees that the critical value of [*i-1*, *i*] in (*u*_[_*_i-1_*_]_ + $\frac{\alpha_{[i]}}{1 - \alpha_{[i]}}$*ε*, *u*_[_*_i_*_]_ - *ε*, *u*_-[_*_i-1_*_,_ *_i_*_]_) is given by *α*_[_*_i_*_]_(*u*_[_*_i_*_]_ - *ε*) + (1 - *α*_[_*_i_*_]_)(*u*_[_*_i-1_*_]_ + $\frac{\alpha_{[i]}}{1 - \alpha_{[i]}}$*ε*) = *α*_[_*_i_*_]_ *u*_[_*_i_*_]_ + (1 -*α*_[_*_i_*_]_) *u*_[_*_i-1_*_]_. Similarly, the critical value of [*i-1*, *i*] in (*u*_[_*_i-1_*_]_ - $\frac{\alpha_{[i]}}{1 - \alpha_{[i]}}$*ε*, *u*_[_*_i_*_]_ + *ε*, *u*_-[_*_i-1_*_,_ *_i_*_]_) is given by *α*_[_*_i_*_]_(*u*_[_*_i_*_]_ + *ε*) + (1 - *α*_[_*_i_*_]_)(*u*_[_*_i-1_*_]_ - $\frac{\alpha_{[i]}}{1 - \alpha_{[i]}}$*ε*) = *α*_[_*_i_*_]_ *u*_[_*_i_*_]_ + (1 -*α*_[_*_i_*_]_) *u*_[_*_i-1_*_]_. ■

It is easy to extend the concept of the critical value of [*i-*1, *i*] to the version of any set of ranks. Indeed, strong Pareto and completeness imply the uniqueness of a critical value ∑_[_*_i_*_]∈[_*_M_*_]_ *w*_[_*_i_*_]_ *u*_[_*_i_*_]_ of [*M*] in any profile *u_N_*, whereas a rank-dependent weight vector *w*_[_*_M_*_]_ is not uniquely determined (Claim 5 guarantees the uniqueness of the weight vector). In fact, for all profiles *u_N_*, a critical value of [*M*] can be written as *inf* {*αu_max_* + (1 - *α*)*u_min_* | *α*∈[0, 1] and ((*αu_max_* + (1 - *α*)*u_min_*)_[_*_M_*_]_, *u_-_*_[_*_M_*_]_) ≻ *u_N_* } where *u_min_* is a minimum and *u_max_* is a maximum in *u*_[_*_M_*_]_. As shown in Claim 5, this critical value can be represented by a rank-weighted sum with the unique rank-dependent weight vector of [*M*]. For convenience, I write *u_N_* ≈_[_*_M_*_]_ *v_N_* if both *u_N_* and *v_N_* have the same critical value of [*M*]. Then, for all profiles *u_N_*, *v_N_* ∈*U ^N^* with *u*_-[_*_i-1_*_,_ *_i_*_]_ = *v*_-[_*_i-1_*_,_ *_i_*_]_, it is shown that the same critical value of [*i-1*, *i*] means the same critical value of [*M*], including [*i*].

(Claim 4) ∀[*i*] ≠ 1, ∀*u_N_*, *v_N_* ∈*U ^N^* with *u*_-[_*_i-1_*_,_ *_i_*_]_ = *v*_-[_*_i-1_*_,_ *_i_*_]_, ∀consecutive [*M*] with [*i*], [*i*-1] ∈ [*M*] if *u_N_* ≈_[_*_i_*_-1,_ *_i_*_]_ *v_N_* , then *u_N_* ≈_[_*_M_*_]_ *v_N_*.

Suppose that$u_{N}\approx_{\left[ i-1, i \right]}(u_{-\left[ i-1, i \right]},2*\lambda)$ and $u_{N}\approx_{\left[ M \right]}(u_{-\left[ M \right]},m*\mu)$ where for all natural numbers *m* and real numbers $\mu$, $m*\mu=(\mu, \ldots,\mu)$ is the *m-*time replications of $\mu$. Then, I will show ${(u_{-\left[ i-1, i \right]},2*\lambda)\approx}_{\left[ M \right]}(u_{-\left[ M \right]},m*\mu)$.

Suppose, contrarily, that ${\left( u_{-\left[ i-1, i \right]},2*\lambda\right)\approx}_{\left[ M \right]}\left( u_{-\left[ M \right]},m*\mu^{'} \right)$ and $\mu^{'}>\mu$.

Consider $\lambda^{*}$ such that ${\left( u_{-\left[ i-1, i \right]},2*\lambda^{*} \right)\approx}_{\left[ M \right]}\left( u_{-\left[ M \right]},m*\mu\right)$. Note that $\lambda^{*}<\lambda$ must be true because $\mu^{'}=\mu$ if $\lambda^{*}=\lambda$ and for all $\mu^{''}\in\left( \mu, \mu^{'} \right), \left( u_{-\left[ i-1, i \right]},2*\lambda^{*} \right)\succ\left( u_{-\left[ i-1, i \right]},2*\lambda\right)\succ\left( u_{-\left[ M \right]},m*\mu^{''} \right)\succ\left( u_{-\left[ i-1, i \right]},2*\lambda^{*} \right)$ if $\lambda^{*}>\lambda$.

Next, for all $\mu^{''}\in\left( \mu, \mu^{'} \right),$let $\lambda^{''}$be such that $\left( u_{-\left[ M \right]},m*\mu^{''} \right)\approx_{[M]}\left( u_{-\left[ i-1, i \right]},2*\lambda^{''} \right).$ Note that $\lambda^{''}\in\left( \lambda^{*}, \lambda\right)$ because of the above argument.

By $u_{N}\approx_{\left[ i-1, i \right]}\left( u_{-\left[ i-1, i \right]},2*\lambda\right)$ and $\lambda^{''}<\lambda$, $u_{N}\succ\left( u_{-\left[ i-1, i \right]},2*\lambda^{''} \right)$.

By $\left( u_{-\left[ M \right]},m*\mu^{''} \right)\approx_{[M]}\left( u_{-\left[ i-1, i \right]},2*\lambda^{''} \right)$, for all $\varepsilon>0$ with $\mu^{''}-\varepsilon>\mu$, $\left( u_{-\left[ i-1, i \right]},2*\lambda^{''} \right)\succ\left( u_{-\left[ M \right]},m*{(\mu}^{''}-\varepsilon) \right)$.

By $u_{N}\approx_{\left[ M \right]}(u_{-\left[ M \right]},m*\mu)$ and $\mu^{''}-\varepsilon>\mu$, $\left( u_{-\left[ M \right]},m*{(\mu}^{''}-\varepsilon) \right)\succ u_{N}$.

Transitivity implies $u_{N}\succ u_{N}$ which is a contradiction.

Thus, $\mu^{'}>\mu\mathrm{is}$false. It is easily proved that $\mu^{'}<\mu$ is also false because of the similar argument. Hence, $\mu^{'}=\mu$.

Finally, suppose that $v_{N}\approx_{\left[ M \right]}(u_{-\left[ M \right]},m*\kappa)$. Then, the above result implies ${(u_{-\left[ i-1, i \right]},2*\lambda)\approx}_{\left[ M \right]}(u_{-\left[ M \right]},m*\kappa)$ because $v_{N}\approx_{\left[ i-1, i \right]}\left( u_{-\left[ i-1, i \right]},2*\lambda\right).$ By the definition of critical values, $\kappa= \mu$ which implies $u_{N}\approx_{\left[ M \right]}v_{N}$.■

(Claim 5) ∀*u_N_* ∈*U ^N^*, ∀[*M*] = {[*i*], [*i-1*], …, [*i-m*]} with a sequence of non-zero thresholds (*α*_[_*_i_*_]_, …, *α*_[_*_i-m_*_]_), $u_{N}\approx_{[M]}$((∑_[_*_j_*_]∈[_*_M_*_]_ *w*_[_*_j_*_]_ *u*_[_*_j_*_]_)_[_*_M_*_]_, $u_{-\left[ M \right]}$) where $w_{[i]}$: $w_{[i-1]}$ : …: $w_{[i-m]}$ = 1: $\frac{{1 - \alpha}_{[i]}}{\alpha_{[i]}}$ : …: $\prod_{j=i, i-1,\ldots,i-m} \frac{1-\alpha_{[j]}}{\alpha_{[j]}}$.

By Claims 3 and 4, $u_{N}\approx_{[M]}$($u_{[i-1]}+\frac{\alpha_{[i]}}{1 - \alpha_{[i]}}\varepsilon$, $u_{[i]}-\varepsilon$, $u_{-\{\left[ i-1 \right], \left[ i \right]\}})\approx_{[M]}$($u_{[i-2]}+\frac{\alpha_{[i]}}{1 - \alpha_{[i]}}\cdot\frac{\alpha_{[i-1]}}{1 - \alpha_{[i-1]}}\varepsilon$, $u_{[i-1]}$, $u_{[i]}-\varepsilon$, $u_{-\left[ i-2, i-1, i \right]}$) $\approx_{\left[ M \right]}$($u_{[i-m]}+\prod_{j=i, i-1,\ldots,i-m} \frac{\alpha_{[j]}}{1 - \alpha_{[j]}}\varepsilon$, $u_{[i-m+1]}$, $u_{[i]}-\varepsilon$, $u_{-\left[ i-m, i-m+1, i \right]}$). This argument means that a *leaky* transfer of well-being from rank [*i*] to rank [*i*-*k*] that maintains the same critical value must be proportional to the ratio $\prod_{j=i, i-1,\ldots,i-k} \frac{\alpha_{j}}{1 - \alpha_{j}}$. Note that these weights are simply their ratios because only relative weights matter. Thus, the critical value ∑_[_*_j_*_]∈[_*_M_*_]_ *w*_[_*_j_*_]_ *u*_[_*_j_*_]_ of [*M*] in *u_N_* is exactly determined in Claim 5. ■

Finally, the generalized leximin rule is obtained by combining the above results. By Claim 5, ∀*u_N_* ∈*U ^N^*, ∀[*M*] = {*i*, *i-1*, …, *i-m*} with a sequence of non-zero thresholds (*α*_[_*_i_*_]_, …, *α*_[_*_i-m_*_]_), the critical value of [*M*] in *u_N_* is given by the rank-weighted utilitarianism defined in [*M*]. If ∃[*i**] with its threshold *α*_[_*_i*_*_]_ = 0, then the critical value of [*M_i_*_*_] containing [*i**] in *u_N_* is lexicographically preferred to that of [*M_i_*] containing [*i*] for all [*i*] > [*i**]. This means ≽ is the generalized leximin rule. ■

The Proof of Theorem 3: Since anonymity and separability implies rank-separability, there exists a unique threshold *α*∈[0, 1] such that∀*u_N_* ∈*U ^N^* with *u*_[_*_i_*_]_ > *u*_[_*_j_*_]_,∀*α' >α*, ((*α' u*_[_*_i_*_]_ + (1 - *α'*)*u*_[_*_j_*_]_ )_[_*_j_*_,_ *_i_*_]_, *u*_-[_*_j_*_,_ *_i_*_]_) ≻ *u_N_* & ∀*α' <α*, *u_N_* ≻ ((*α' u*_[_*_i_*_]_ + (1 - *α'*)*u*_[_*_j_*_]_ )_[_*_j_*_,_ *_i_*_]_, *u*_-[_*_j_*_,_ *_i_*_]_) by the similar proof of Claim 1 in Theorem 1 (The similar logic easily proves the similar results of Claims 1-5 in Theorem 1 *without imposing Pigou-Dalton transfer*). Note that a threshold *α* is independent of any rank because the ranks of *u*_[_*_i_*_]_ and *u*_[_*_j_*_]_ can be any possible combination of ranks owing to separability. If *α* = 0 (resp. 1), it must be leximin (resp. leximax). Consider the case *where α*∈(0, 1). For all [*i*] and [*j*], *w*_[_*_i_*_]_: *w*_[_*_j_*_]_ = 1: $\left( \frac{1 - \alpha}{\alpha} \right)$*^i^*^-^*^j^* based on the logic of Claim 5 in the proof of Theorem 1. In addition, separability requires [*i*] and [*j*] to be independent of any differences between ranks, that is, *w*_[_*_i_*_]_: *w*_[_*_j_*_]_ = 1: $\left( \frac{1 - \alpha}{\alpha} \right)$*^i^*^-^*^j^* = 1: $\left( \frac{1 - \alpha}{\alpha} \right)$. Thus, *α* must be 0.5, implying that a social welfare ordering is weak utilitarian. ■
