# Supplementary material for: Old and new joint characterizations of leximin and variants of rank-weighted utilitarianism
Source: PLoS One. 2024 Jan 2;19(1):e0296351. doi: 10.1371/journal.pone.0296351 (PMC10760868; doi:10.1371/journal.pone.0296351)
Supplement: S2 Appendix — (DOCX) [file pone.0296351.s002.docx]

**Appendix 2.**

This appendix provides the definitions of the axioms that are not formally explained in the paper.

The perfect equity axiom was proposed by Tungodden [22]. It says that if two profiles *u_N_* and *v_N_* are Pareto non-comparable and all individuals enjoy the same well-being level in *u_N_* but *v_N_* is not so, then *u_N_* is strictly better than *v_N_*.

**Perfect Equity**. $\forall u_{N},v_{N}\in U^{N}$ with $u_{N}≯v_{N} \& v_{N}≯u_{N}$, if $\forall i, j\in N, u_{i}=u_{j}$and $\exists i, j\in N, v_{i}\neq v_{j}$, then $u_{N}\succ v_{N}$.

Almost co-cardinality was introduced by Gevers (1979). It says that if positive affine transformations are applied to profiles $u_{N}$ and $v_{N}$ so as not to change the ranks of individuals within these profiles (these affine transformations allow the intercept $a_{i}$ to be different among individuals, while the positive coefficient $b$ must be common), the social evaluation should be invariant with respect to these transformations.

**Almost Co-cardinality.** $\forall u_{N},v_{N}\in U^{N},\forall a_{1}, \ldots,a_{n}\mathbb{\in R,\forall}b\in\mathbb{R}_{++},\forall$strictly increasing numerical functions $\varphi, u_{N}≽v_{N}\leftrightarrow(a_{i}+bu_{i})_{i\in N}≽(a_{i}+bv_{i})_{i\in N}$ where $\forall i\in N, \varphi(u_{i})=a_{i}+bu_{i}$ and $\varphi(v_{i})=a_{i}+bv_{i}$.

The original version of strict composite transfer was proposed by Shorrocks and Foster [26]. The axiom considers *composite* *transfers* which consist of a transfer of the same amount $\varepsilon$ from *i* to *j*, combined with a simultaneous transfer of the same amount $\delta$ from *k* to *l*, keeping the same ranks among well-being distributions through this composite transfer. Suppose that the transfer from *j* to *i* is *progressive* while that from *k* to *l* is *regressive*, and both *i* and *j* are poorer than *k* and *l*. Then, strict composite transfer demands this composite transfer should increase social welfare.

**Strict Composite Transfer**. $\forall u_{N},v_{N}\in U^{N}, \forall\varepsilon, \delta\in\mathbb{R}_{++}$, if $\exists i, j, k,l\in N,u_{l}=v_{l}+\delta{\geq v}_{k}-\delta=u_{k}\geq v_{i}-\varepsilon=u_{i}\geq u_{j}=v_{j}+\varepsilon$ and $\forall hN\setminus\{i, j, k, l\}, u_{h}=v_{h}$, then $u_{N}\succ v_{N}$.

Finally, strict Pigou-Dalton transfer simply requires that any progressive transfer of the same amount $\varepsilon$ from *i* to *j* should increase social welfare.

**Strict Pigou-Dalton Transfer**. $\forall u_{N},v_{N}\in U^{N}, \forall\varepsilon\in\mathbb{R}_{++}$, if $\exists i, j\in N, v_{i}-\varepsilon=u_{i}\geq u_{j}=v_{j}+\varepsilon$ and $\forall kN\setminus\{i, j\}, u_{k}=v_{k}$, then $u_{N}\succ v_{N}$.
